# Food perception in Orthorexia nervosa: Craving or avoidance?

**DOI:** 10.1007/s40519-026-01826-8

**Published:** 2026-02-24

**Authors:** Marilena Aiello, Massimiliano Alberto Umiltà, Giovanni Ottoboni, Alessia Tessari

**Affiliations:** 1 Department of Psychology, University of Bologna, Viale Berti Pichat 5, 40127 Bologna, Italy; 2 Department of Humanities and Social Sciences, Universitas Mercatorum, Rome, Italy

**Keywords:** Attentional bias, Reward responsiveness, Eating disorders, addiction, healthiness

## Abstract

Orthorexia nervosa (ON) is characterised by an excessive preoccupation with eating healthy foods. This disorder shares similarities with various pathological conditions, including anorexia nervosa and addictive behaviours. This research aimed to determine whether ON is primarily driven by a fear of unhealthy foods, similar to anorexia nervosa, or craving for healthy foods, akin to the patterns observed in addictive disorders. In an online study (Study 1), participants (n = 166 adults, mean age = 24.8 years, SD = 7.6 years, 48.8% female) reported their liking, wanting, and frequency of consumption of 20 healthy and 20 unhealthy foods. Additionally, they completed the Düsseldorf Orthorexia Scale, while BMI, hunger level, and risk of eating disorders were collected. In Study 2, participants (n = 73 adults, mean age = 23.4 years, SD = 3.5 years, 37% female) completed questionnaires on ON and the risk of eating disorders, as well as a visual probe task with images of healthy and unhealthy foods. Eye movements were also recorded for a subset of participants in the laboratory. The results suggest that individuals with more pronounced levels of orthorexic features exhibit decreased sensitivity to food rewards and an attentional avoidance of unhealthy foods. This indicates that ON behaviours may be driven by a fear of unhealthy foods. These results underscore the importance of elucidating the role of attentional and motivational mechanisms in ON and their clinical implications.

## Introduction

Orthorexia nervosa (ON) is a condition characterised by an obsessive concern with eating healthy foods [1, 2]. Individuals with ON express concern about meal quality and composition and engage in rigid eating behaviour in an attempt to achieve health through "food purity". They follow severe dietary rules that result in excessive avoidance of "unhealthy" foods. For instance, cases of patients eating only seeds or unprocessed, organic plant-based foods, or even only animal products, have been described [3]. Furthermore, they devote significant time—approximately 4–5 h daily—to managing (thinking, choosing, controlling) food sources and preparing meals [3]. Eating foods considered "unhealthy" causes feelings of guilt, disgust, fear of becoming ill, and self-punishment behaviours. Conversely, the sense of control over food intake is perceived positively and reinforces this behavior, which, in the long term, can affect health as well as social relationships, leading to nutrient deficiency, malnutrition, and social isolation [4]. To date, the prevalence of ON behaviours has been reported to range from 7 to 57% in the general population, from 29 to 34.9% among Italian university students and higher in some “risk groups,” such as healthcare professionals and dietitians (general population: [5], university students: [6], dietitians: [7]).

Although ON is not currently recognized as an official disorder in the Diagnostic and Statistical Manual of Mental Disorders (DSM-5), and its conceptualisation has been debated (e.g., [8, 9]), it is increasingly understood to overlap with several established psychiatric disorders. For instance, ON is believed to share characteristics with anorexia nervosa, including perfectionism, restrictive eating patterns, food-related preoccupation (e.g., [10]), and obsessive–compulsive disorders, as individuals with ON exhibit intrusive thoughts (similar to obsessions) about food, fear of health contamination and impurities associated with distress [11]. ON has also been proposed to belong to the ‘‘behavioural’’ addiction disorder category, together with pathological gambling, internet addiction, and overtraining syndrome [12]. Similarly to these conditions, ON appears to be characterised by repetitive dysfunctional habits that induce a progressive impairment of affective, working, and social spheres, and by loss of control over behaviour despite its negative consequences [13]. Therefore, additional research on, particularly concerning its similarities and differences with these conditions, is essential for enhancing our understanding of the cognitive processes underlying ON and developing more effective diagnostic and therapeutic strategies.

Multiple forms of psychopathology are associated with dysregulation of attentional processes [14] and specifically attentional bias (AB), which involves the selective allocation of attention to salient stimuli. AB to negative disease-salient information perceived as threatening has been described in anxiety disorders (e.g., [15]), and it is believed to reflect high levels of worry. For instance, individuals who fear spiders or snakes show both an attentional bias toward words or pictures signalling these stimuli (for example, [16]) and a time course characterised by an early attentional bias toward threat stimuli, followed by intentional avoidance of these same stimuli [17]. Similar patterns have been observed with caloric food or body pictures in anorexia nervosa [18]. However, in addiction disorders, AB may reflect craving and hedonic motivation [19, 20]. Craving is described as an intense, irresistible desire to engage in a specific behavior and is the result of learning processes during which relevant cues become associated with the rewarding features of addictive behaviour [21]. An AB for drugs or disorder-related stimuli has been observed in individuals with drug addiction [19], problem gambling [22], internet gaming disorders [23], and binge eating disorders [18].

To date, only two studies have investigated AB for healthy/unhealthy food cues in orthorexic individuals [24, 25], and both suggest that an attentional bias toward healthy food words characterises ON. In the first study, ON tendencies were associated with increased attentional preference for healthy food-related words in a modified Stroop task [24]. Similar findings were obtained in a second study, in which the allocation of attention to healthy/unhealthy food-related words was evaluated using a Dot Probe task [25]. In the dot-probe task, a pair of stimuli is presented simultaneously on the screen. In target trials, one of these stimuli is considered relevant (healthy food words such as *salad* or unhealthy food words such as *salami*), while the other is neutral (for instance, *salary*). Immediately after the stimulus pair is removed, a probe (a dot) is presented at one of the two stimulus locations, and the participants are asked to indicate the location of the probe. Participants are assumed to react faster to a stimulus that appears in a location where their attention is already focused than to a stimulus appearing in an unattended location. Using this task in a sample of vegans/vegetarians, Albery et al. [25] confirmed a significant correlation between attentional bias for healthy food words and ON tendencies. Despite these interesting results, no information on the time course or hedonic motivation toward healthy/unhealthy foods was provided in these studies.

In this research, we aimed to investigate both reward responsiveness and attentional bias toward healthy and unhealthy food pictures in individuals with varying levels of ON through two distinct studies. Study 1 examined the ratings of liking and wanting for healthy and unhealthy foods. Liking and wanting are two essential components of the brain's reward system. "Liking" refers to the pleasurable experience associated with consuming food, driven by hedonic hotspots in the limbic brain structures. In contrast, "wanting" involves incentive salience—a motivational process that enhances the appeal of palatable foods and can trigger cravings. This process is regulated by dopamine-driven mesocorticolimbic networks [26]. Study 2 investigates AB toward healthy and unhealthy food cues through a dot-probe task. In a subsample of participants in Study 2, we employed an eye-tracking methodology to record gaze patterns during the task. Attentional engagement (early processing) can be assessed by measuring the proportion of trials in which the first saccade is made toward a stimulus. Similarly, we recorded the total gaze duration on a stimulus as a measure of maintained attention (late processing) [27]. Both indices can be considered as indicators of visual attentional bias. If Orthorexia is akin to addiction disorders, increased wanting for healthy foods and an AB for these stimuli should be observed. Conversely, consistent with patterns observed in anorexia nervosa, individuals with ON may exhibit negative evaluations of unhealthy food compared to healthy food pictures on an explicit level, and aberrant attentional processing toward them, because of their preoccupation with and/or fear of food being considered unhealthy. Their preoccupation may be represented by initial engagement, followed by avoidance, as observed in anorexia nervosa.

## Study 1. Reward responsiveness toward healthy and unhealthy food cues and ON

### Participants and procedure

Participants were eligible for inclusion if they met the following criteria: (1) aged 18 or older, (2) native Italian speakers, (3) absence of neurological/psychiatric disease, and 4) absence of diabetes and food restrictions (e.g., vegetarianism, allergy, or others). A total of 166 eligible participants completed the survey on the Qualtrics platform (age_mean_ 24.8 years, age_SD_ = 7.6, age range = 18–60; 48.8% female). Participants were recruited via social media platforms and university mailing lists. The study ads informed participants about the study's aim and provided a link to complete the study on Qualtrics (www.qualtrics.com). The participants were not compensated for their participation and were informed that they were free to withdraw from participation at any time. The Ethics Committee of the University of Bologna approved the study (protocol no. 0062640 of 08/03/2023). After obtaining informed consent, the participants provided demographic data, health status information, and dietary habits via a self-report questionnaire. We asked them to report their hunger level (from 0 = not hungry to 7 = extremely hungry), hours since their last meals and their weight and height to compute their body mass index (BMI). Participants then completed an explicit evaluation task assessing reward responsiveness toward healthy and unhealthy foods, and questionnaires on ON and eating disorder risk.

### Task and questionnaires

**Fig. 1:**
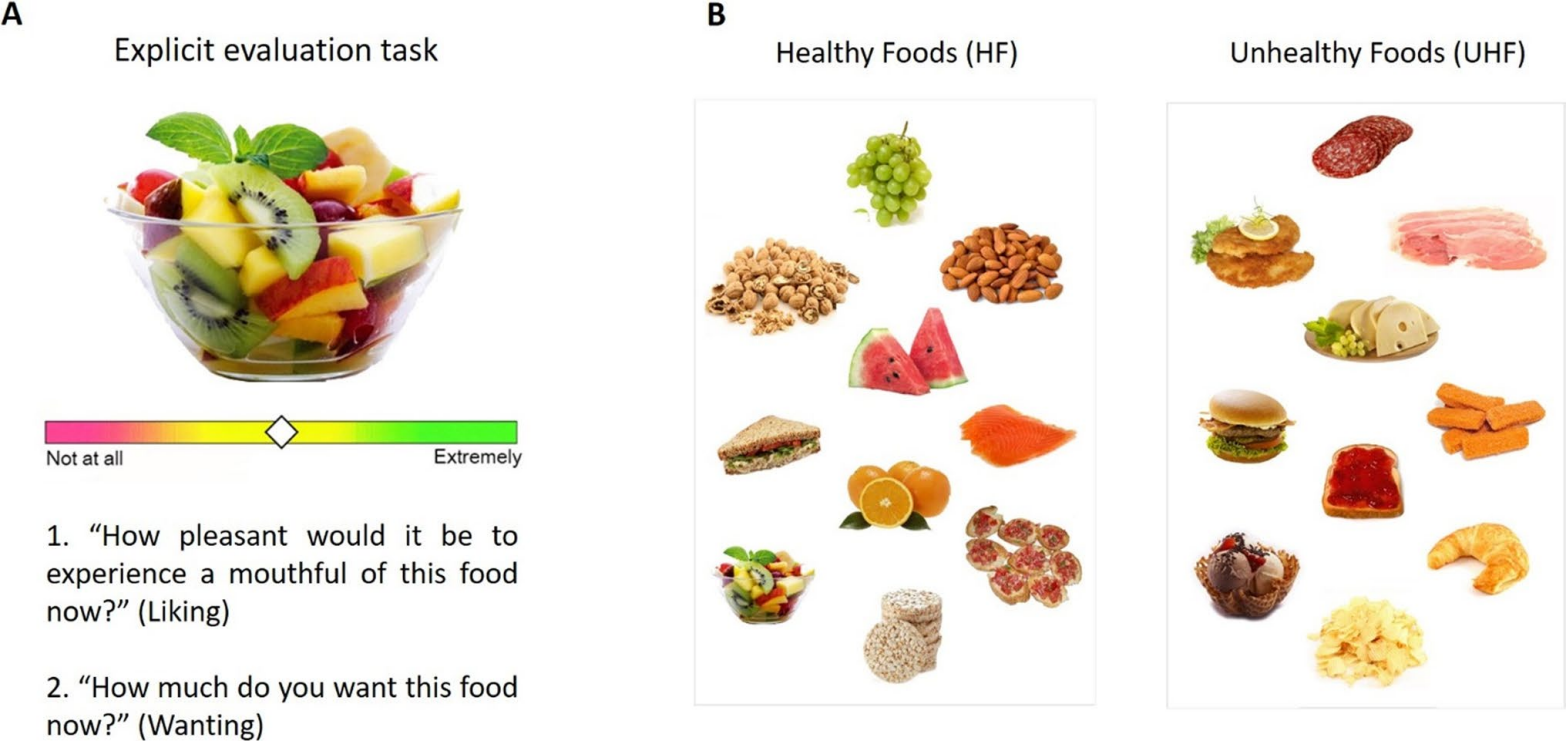
A. Example of explicit liking and wanting ratings. B. Pictures of food used in the study

*Explicit Evaluation of Foods*. This task was aimed at investigating reward responsiveness to healthy and unhealthy foods. In this task, 20 pictures [10 healthy (HF) and 10 non-healthy (NHF) foods] were presented and participants were asked to respond to the following questions: (1) “How pleasant would it be to taste a morsel of this food now?” (Liking); (2) "How much do you want this food now?" (Wanting). In addition, we presented two additional questions: (3) "How frequently do you eat this food?" and (4) "How healthy is it?" Participants indicated their responses on a 100-point visual analogue scale anchored at each end with “not at all” and “extremely” (for a similar procedure, see [28]). Food pictures were taken from FRIDA and FoodPics databases [29, 30]. Healthy and unhealthy food images were selected through an online questionnaire filled out by an independent sample of 20 participants (six females, age_mean_ 20.3 age_SD_ 1.9). Importantly, healthy and unhealthy foods were matched for caloric content [t (18) = 0.82, *p* = 0.42], frequency of consumption [t (18) = − 1.98, *p* = 0.06], and palatability [t (18) = 1.54, *p* = 0.14], whereas they significantly differed in perceived healthiness [t (18) = − 8.05, *p* = 0.00]. See Fig. 1.

*Questionnaires.* The *Düsseldorf Orthorexia Scale* (DOS) [31, 32] evaluates orthorexic tendencies and is composed of 10 items on a 4-point Likert scale, from 1 (it does not correspond to my behaviour at all) to 4 (it corresponds well to my behaviour). The maximum score is 40, with higher scores indicating more pronounced orthorexic behaviour. A score ≥ 30 is considered indicative of the presence of ON, while a score between 25 and 29 indicates the risk of ON. The Eating Attitudes Test (EAT-26) [33, 34]) assesses the risk of eating disorders. Responses to questions 1–25 are rated on a four-point scale: "always" is assigned 3 points, "usually" is assigned 2 points, "often" is assigned 1 point, and "sometimes," "rarely," or "never" receives 0 points. Reverse scoring is applied to item 26. A score ≥ 20 is considered indicative of disordered eating tendencies.

### Statistical analyses

Participants were categorized into two groups based on the median DOS score (median value = 20), with 83 participants in the HighON and 83 in the LowON groups. Normality was assessed using the Shapiro–Wilk test. Parametric data were analysed using 2 × 2 repeated-measures ANOVAs, where the type of food (two levels: HF and NHF) was included as a within-subject factor and the group of participants (two levels: HighON and LowON) as the between-subject factor. Non-parametric variables were analysed using the Mann–Whitney U and the Wilcoxon signed-rank tests. Pearson correlation coefficient was used to examine the relationships between DOS and food ratings. All analyses were performed using Statistica software (Statsoft, Tulsa, USA). Violin plots were made using the online platform SRplot [35].

### Results

#### Sample characteristics

Overall, the average DOS score was 19.5 (SD = 5.4), with a range of 10–38. Using the established DOS cut-off criteria (ON: ≥ 30; borderline ON: 25–29), 4.2% of participants were classified as ON, 9.7% as borderline ON, and 86.1% as non-ON. The average EAT-26 score was 8.1 (SD = 8.7), with a range of 0–44. The participants’ BMI ranged between 17 and 31.3 (Mean = 22.7, SD = 2.8). Finally, participants reported a mean hunger score of 2.8 (SD = 1.9), with a range of 0–7.

#### Explicit ratings

The HighON and LowON groups did not differ significantly in terms of BMI (U = 3309, Z = 0.44, *p* = 0.66), hunger level [U = 3145, Z = 0.96, *p* = 0.33], or hours since their last meal (U = 3218, Z = 0.73, *p* = 0.46). However, a significant difference was observed in EAT-26 scores (U = 1651, Z = -5.79, *p* < 0.001).

The ANOVA on liking ratings revealed a significant interaction between group and food category [F (1, 164) = 10.34, *p* = 0.001], while other main effects were not significant: group [F (1, 164) = 2.46, *p* = 0.11], category [F (1, 164) = 2.29, *p* = 0.13]. Post-hoc analyses showed that individuals in the HighON group liked unhealthy foods less than healthy foods (*p* = 0.001), while no such difference was found in the LowON group (*p* = 0.23). Additionally, individuals in the HighON group liked unhealthy foods less than those in the LowON group (*p* = 0.02), whereas no significant difference emerged for healthy foods (*p* = 0.90). The analysis of wanting ratings revealed that overall wanting was higher for healthy foods than unhealthy foods [F (1, 164) = 5.3, *p* = 0.02] while the main group effect was not significant [F (1, 164) = 0.18, *p* = 0.6]. Moreover, a significant interaction between group and food category also emerged [F (1, 164) = 7.03, *p* = 0.008]. Post-hoc analyses indicated that individuals in the HighON group wanted unhealthy food less than healthy food (*p* < 0.001), while no such difference was observed in the LowON group (*p* = 0.81). No differences between the HighON group and the LowON group were found in wanting ratings (*ps* > 0.24).

**Fig. 2:**
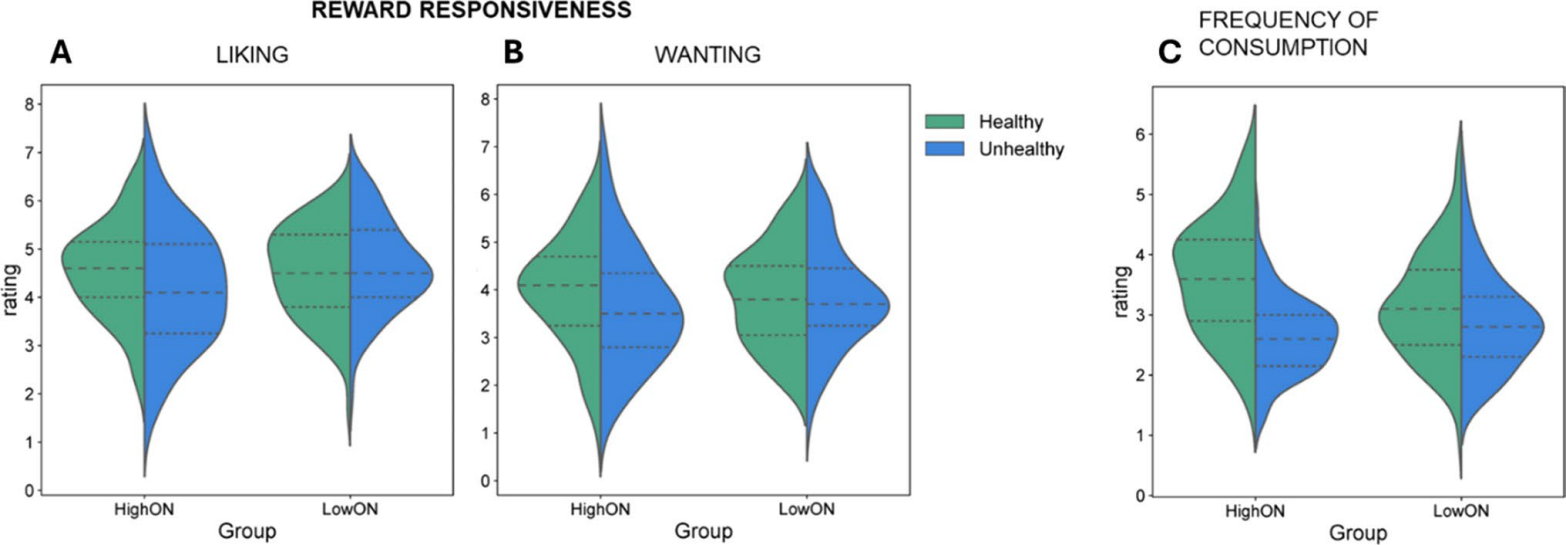
Violin plots illustrating the distributions of ratings for Liking (**A**), Wanting (**B**), and Frequency of Consumption (**C**) in the HighON and LowON groups, separately for healthy and unhealthy food images. The width of each violin reflects the density of the data. The central horizontal line indicates the median, and the dotted lines denote the interquartile range. Whiskers extend to the minimum and maximum observed values

Moreover, participants reported consuming healthy foods more frequently than unhealthy foods (*p* < 0.001). Individuals in the HighON group reported consuming healthy foods more frequently than those in the LowON group (U = 2407.5, Z = − 3.34, *p* < = 0.01), whereas no difference in the consumption of unhealthy foods was found between the two groups (U = 2944.5, Z = 1.61, *p* = 0.10). Finally, as expected, healthy foods were rated as healthier than unhealthy foods (*p* < 0.001), and no significant difference emerged in the perceived healthiness of the two food categories between the groups [healthy foods: U = 3402, Z = 0.13, *p* = 0.89; unhealthy foods: U = 3420, Z = 0.07, *p* = 0.93]. Pearson correlation results are shown in Table 1 (Fig. 2).

**Table 1:** Pearson correlation between DOS scores and explicit ratings

	HF-Liking	UHF-Liking	HF-Wanting	UHF-Wanting	HF-Consumption	UHF-Consumption
DOS	0.05	− 0.18	0.12	− 0.12	0.37	− 0.14
	*p* = 0.48	*p* = 0.02	*p* = 0.13	*p* = 0.13	*p* = 0.00	*p* = 0.07

Explicit liking and wanting ratings were highly correlated for both healthy foods (r = 0.85, *p* < 0.001) and unhealthy foods (r = 0.79, *p* < 0.001).

## Study 2: AB toward healthy and unhealthy foods in ON

### Participants and procedure

This study was conducted using two independent samples. The first sample performed the task online, the second sample was tested in the laboratory, and eye movements were recorded. The inclusion criteria for both samples were the same as in Study 1, except for age. Given the attentional nature of the task, only participants aged between 18 and 45 years were included. Sample 1 was composed of 42 participants (age_mean_: 23.1, age_SD_ = 4.3, 45% female), whereas sample 2 was composed of 31 participants (age_mean_: = 23.9, age_SD_ = 1.8, 29% female). None of the participants was compensated for their participation, and they were informed that they were free to withdraw from participation at any time. They provided informed consent to participate in the study, which was in line with the Declaration of Helsinki and approved by the Ethics Committee of Bologna University (Protocol no. 0062640 of 08/03/2023). Regarding the online procedure, after obtaining informed consent, participants provided information about their demographic characteristics, health status, and eating patterns by filling out a questionnaire. Participants then completed a dot-probe task delivered over the Internet using the free software Jatos [36]. Finally, they completed questionnaires on ON and the risk of eating disorders. Regarding laboratory procedures, we began the session by administering the dot-probe task and measuring eye movements. We then asked the participants to complete the two questionnaires.

### Task and questionnaires

**Fig. 3:**
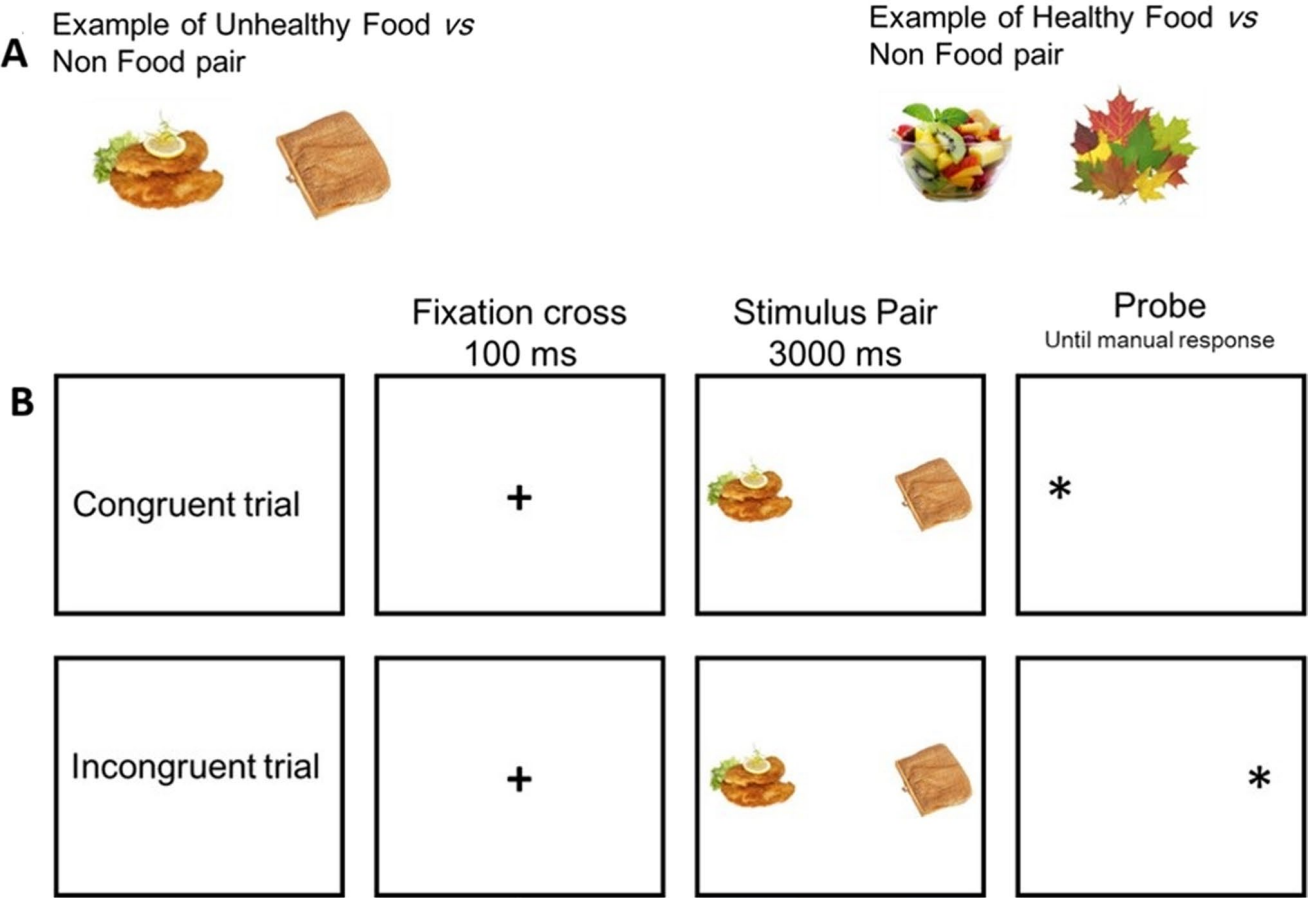
**A**. On the left, a pair of unhealthy foods versus non-food is shown. On the right, a pair of healthy foods versus non-foods. **B**. An example of congruent and incongruent trials

*Dot-Probe Task.* The task followed a procedure similar to the one employed by van Ens et al. [37]. Initially, a fixation cross was displayed in the centre of the screen for 100 ms, followed by the presentation of a picture pair on either side of the centre for 500 ms. Afterwards, a dot (1 × 1 degrees of visual angle) appeared either on the left or right side, and participants were required to indicate the dot's location by pressing the "S" key if it was on the left and the "L" key if it was on the right. A total of 30 pairs of images were used, with 20 being critical pairs (10 healthy foods versus non-food and 10 unhealthy foods versus non-food) and 10 being filler pairs (presenting two non-food pictures). Each pair was presented four times, resulting in a total of 120 trials. The trials were divided into two blocks of 60 trials, separated by a short break, with one block containing images of healthy foods and the other containing images of unhealthy foods. Blocks were counterbalanced across subjects. The position of the probe was counterbalanced. The food pictures used in this study were the same as those used in study one, while the non-food pictures were obtained from the FRIDA and FoodPics databases [29, 30]. Food and Non-food stimuli in the critical pairs were matched as closely as possible for colour and complexity (see Fig. 3 for an example).

In the lab version, participants were seated 64 cm from the screen. Reaction time (RT) data were collected using Matlab software, while a Tobii eye tracker was used to record eye movements.

Regarding the RT data, incorrect responses and RTs deviating by more than two standard deviations from the participant's mean were excluded from subsequent analysis. Three indices were calculated. *RT bias scores* were calculated by subtracting RTs of congruent trials (probe replaced the food image) from RTs of the incongruent trials (probe replaced the control image). Positive values indicate attention bias towards food images; negative values indicate attention bias away from food images and towards control images [37]. Regarding eye movement data, a fixation was defined as a period lasting at least 100 ms in which no saccades or blinks occurred [20]. *Direction bias* was calculated as the percentage of the total initial fixations on food. A score above 50% indicates a direction bias towards food, whereas a score below 50% indicates a direction bias away from food. Direction bias is considered a measure of *initial attentional orientation*. Gaze duration bias, which is considered a measure of *maintained attention*, was calculated using the average gaze duration to a food image across all trials as a proportion of the average gaze duration to all images (food and control). A duration bias score of > 0.5, 0.5, or < 0.5 represents maintained attention to food pictures, no bias and maintained attention to control images, respectively (see [37, 38]).

#### Questionnaires

Participants completed the DOS and the EAT-26 as in Study 1.

### Statistical analyses

As no significant differences were found in reaction times (RTs) between individuals who performed the task online and those who did so in the laboratory for healthy congruent trials, healthy incongruent trials, unhealthy congruent trials, and unhealthy incongruent trials (*ps* > 0.09), the data from both groups were combined for the RT analysis. The data were then divided into two groups based on the median DOS value (median value = 19), with 37 participants in the HighON group and 36 in the LowON group. A similar approach was used for analysing gaze data, where the data were divided into two groups based on the median DOS value (median value = 19), with 16 participants in the HighON group and 15 in the LowON group. Since the variables were not normally distributed, as assessed by the Shapiro–Wilk test, the Mann–Whitney U test and the Wilcoxon signed-rank test were used for data analysis. Spearman correlation analyses were conducted to examine the relationship between ON and RT bias, gaze direction bias, and gaze duration bias. All analyses were performed using Statistica software (StatSoft, Tulsa, USA). The bar charts were constructed using the online platform SRplot [35].

### Results

#### Reaction time data

##### Sample characteristics

The new sample was composed of 73 participants. In the pooled sample, the average score on the DOS was 19.7 (SD 5.7), with a range of 10–38. Using the established DOS cut-off criteria (ON: ≥ 30; borderline ON: 25–29), 5.5% of participants were classified as ON, 12.3% as borderline ON, and 82.2% as non-ON. Furthermore, the average score on the EAT-26 was 6.5 (SD 7.3), with a range of 0–31. In the subsample of participants who completed the dot-probe task with eye-tracking, the mean DOS score was 18.8 (SD = 4.5), with scores ranging from 12 to 28. Based on established cut-off criteria, 12.9% of participants were classified as borderline ON.

Furthermore, the average score on the EAT-26 was 4.3 (SD 4.6), with a range of 0–19. Table 2 presents the reaction times and bias indices for all participants and separately for the two groups (HighON and LowON) for both healthy and unhealthy trials.

**Table 2:** Reaction times (in ms) and bias indices

	Healthy food (HF)		Unhealthy food (UHF)	
	Mean	SD	Mean	SD
*n* = *73*				
RT Congruent trials (ms)	427.2	83.2	415.8	77.9
RT Incongruent trials (ms)	429.3	94.9	427.1	96.7
RT bias score	2.09	42.1	11.2	43.1
*HighON*				
RT Congruent trials (ms)	411.3	71.9	407.7	80.1
RT Incongruent trials (ms)	405.7	72.2	407.6	78.8
Bias index score	− 5.6	37.2	− 0.2	25.6
*LowON*				
RT Congruent trials (ms)	443.7	91.6	424.2	76
RT Incongruent trials (ms)	453.7	109.5	447.1	109.8
Bias index score	10	45.7	23	53.6

In trials presenting healthy foods, there was no significant difference between congruent and incongruent trials for either the HighON or LowON groups (*ps* > 0.30). In both groups, the bias scores did not differ significantly from zero (*ps* > 0.30). Individuals in the HighON group were faster than those in the LowON group in both congruent and incongruent trials (Congruent: U = 480.5, Z = -2.04, *p* = 0.04; Incongruent: U = 428.5, Z = -2.62, *p* = 0.008). No significant difference emerged between the two groups in the bias index score (U = 563.5, Z = − 1.13, *p* = 0.25).

**Fig. 4:**
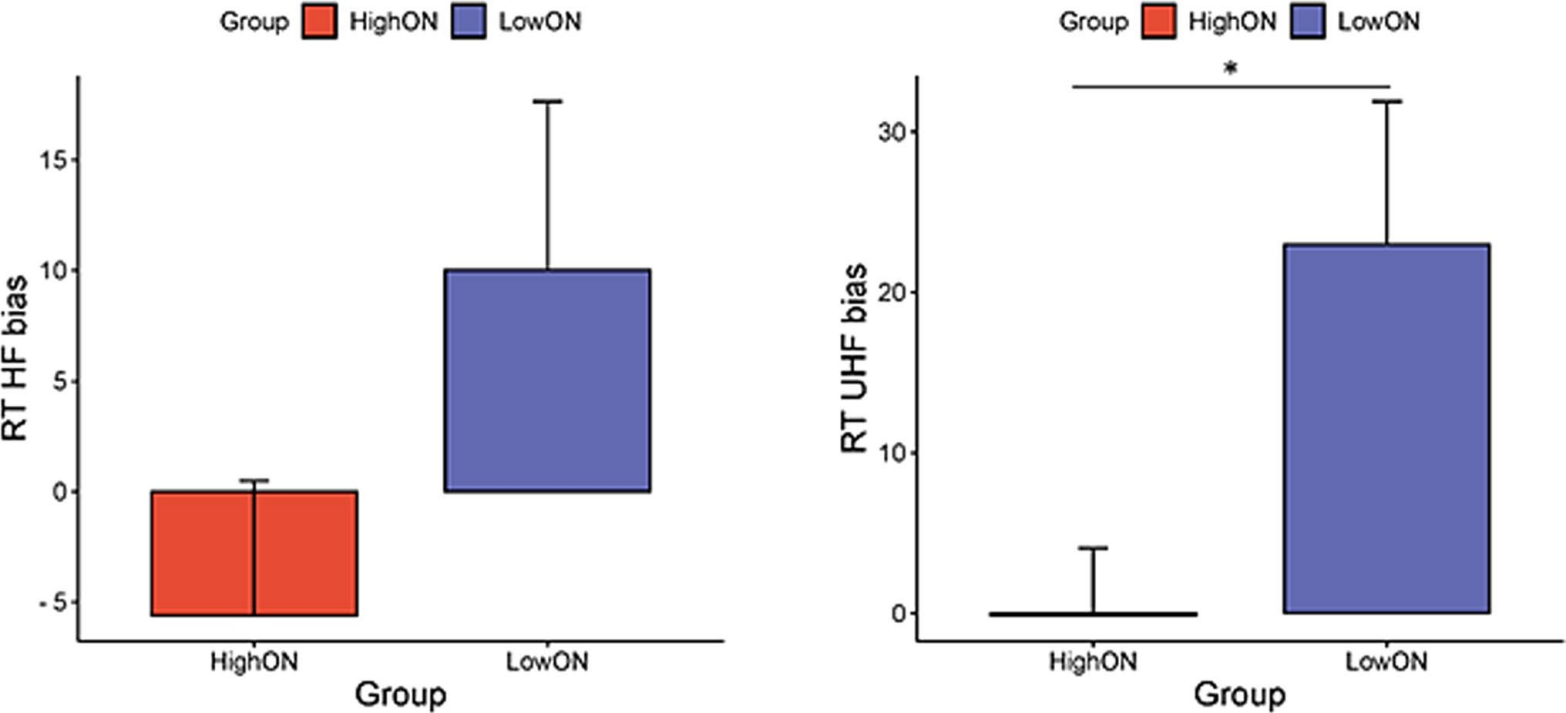
Bar charts with standard error bars displaying RT bias for healthy (HF) and unhealthy (UHF) food in individuals with HighON and LowON

In trials presenting unhealthy foods, individuals in the LowON group were slower in incongruent trials compared to the congruent ones (*p* = 0.007) and exhibited a bias score significantly different from zero (*p* = 0.007), indicating an attentional bias (AB) toward unhealthy food items. In contrast, individuals in the HighON group did not show a significant difference between congruent and incongruent trials (*p* = 0.9), and the bias score did not differ from zero (*p* = 0.92). Individuals in the HighON group were faster than those in the LowON group in the incongruent trials (U = 443.5, Z = − 2.45, *p* = 0.01), but no difference emerged in the congruent trials (U = 529.5, Z = − 1.5, *p* = 0.13). Notably, a significant difference in bias scores emerged between the HighON and LowON groups (U = 480.5, Z = − 2.04, *p* = 0.04). See Fig. 4**.**

No correlations were found between DOS scores and RT bias scores for unhealthy foods (rho = -0.20, *p* = 0.08) and RT bias scores for healthy foods (rho = − 0.07, *p* = 0.53).

#### Gaze bias

Gaze direction and duration bias scores are presented in Table 3 for all participants and separately for the HighON and LowON groups.

**Table 3:** Gaze direction and duration bias scores

	Healthy Food (HF)		Unhealthy Food (UHF)	
	Mean	SD	Mean	SD
*n* = *31*				
Gaze direction bias	49.9	6.0	49.3	6.4
Gaze duration bias	0.51	0.1	0.52	0.1
*HighON*				
Gaze direction bias	50.8	6.1	51.3	6.5
Gaze duration bias	0.52	0.1	0.53	0.1
*LowON*				
Gaze direction bias	49.0	5.8	47.3	5.7
Gaze duration bias	0.51	0.05	0.52	0.04

**Fig. 5:**
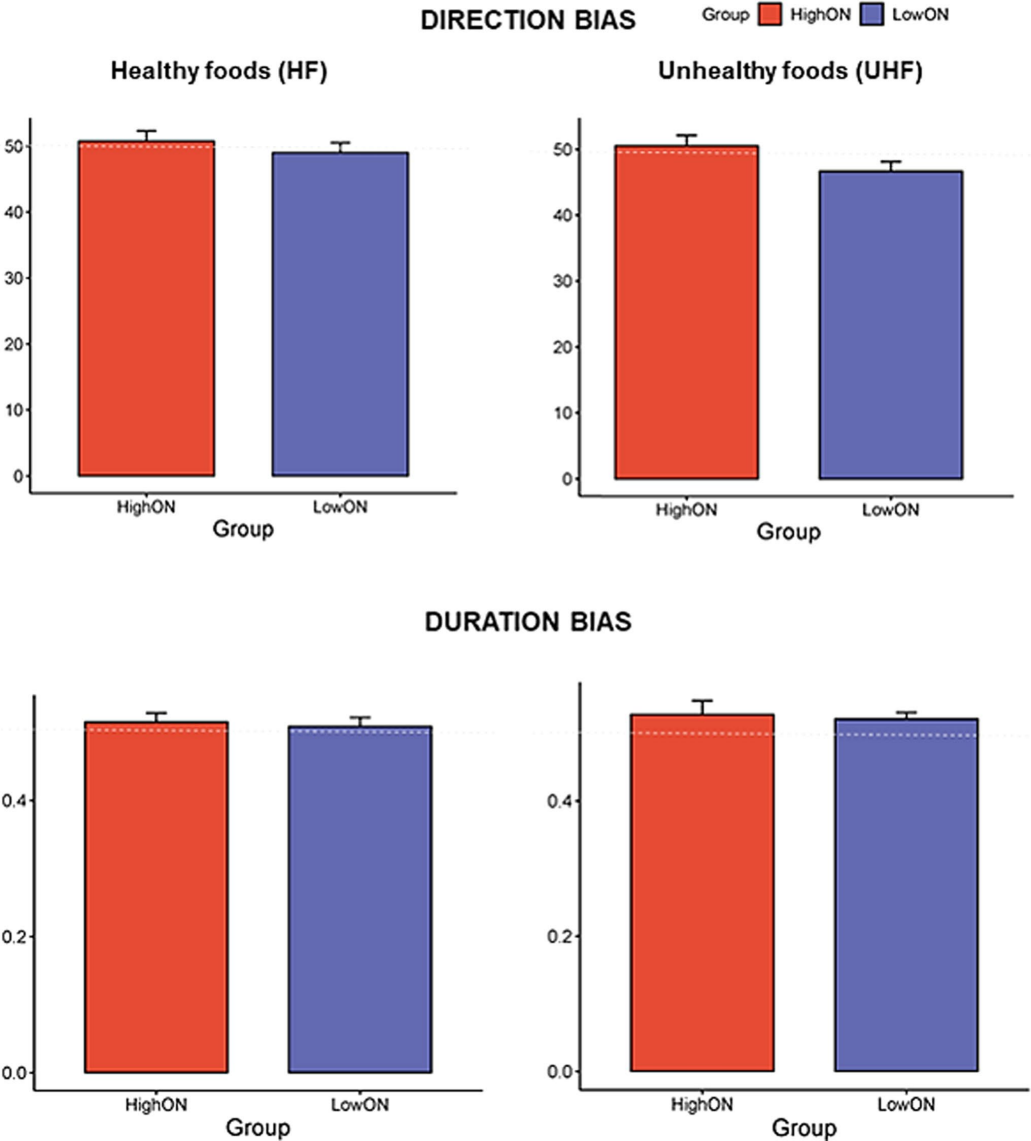
Bar charts with standard error bars displaying Direction and Duration bias for healthy (HF) and unhealthy (UHF) food in individuals with HighON and LowON

For gaze direction bias, individuals in the LowON group did not show any significant difference between healthy and unhealthy foods (*p* = 0.42). Wilcoxon matched-pairs test analysis revealed that the direction bias scores for healthy and unhealthy food images were not significantly different from a test score of 50% (HF: *p* = 0.70, UHF: *p* = 0.10). Similarly, the HighON group did not show any significant difference between healthy and unhealthy foods in gaze direction bias (*p* = 0.67), and the direction bias scores were not significantly different from a test score of 50% (HF: *p* = 0.93, UHF: *p* = 0.50). No difference emerged between groups for either healthy food (U = 109.5, Z = 0.41, *p* = 0.68) or unhealthy food (U = 75, Z = 1.78, *p* = 0.07). See Fig. 5**.**

For gaze duration bias, no significant difference emerged between healthy and unhealthy food images in individuals in the LowON group (*p* = 0.39). Furthermore, the duration bias score for healthy food images was not significantly different from a test score of 0.5 (*p* = 0.53), while the duration bias score for unhealthy food images was significantly different from 0.5 (*p* = 0.049), indicating that participants in this group spent more time looking at unhealthy images compared to non-food images. In individuals in the HighON group, no significant difference was found between healthy and unhealthy gaze duration bias (*p* = 0.16), and the duration bias scores did not differ significantly from a test score of 0.5 (HF: *p* = 0.46, UHF: *p* = 0.25). When comparing the two groups, no significant differences were found for both healthy (U = 114 Z = 0.23 *p* = 0.81) and unhealthy food (U = 108 Z = -0.47 *p* = 0.63). See Fig. 5

A Spearman correlation revealed a significant correlation between DOS and gaze direction bias for unhealthy foods. See Table 4.

**Table 4:** Correlation between DOS scores, Gaze Direction and duration bias

Spearman Correlations				
			rho	*p*
DOS	–	Gaze Direction bias HF	0.06	0.73
DOS	–	Gaze Direction bias UF	0.41	0.02
DOS	–	Gaze Duration bias HF	− 0.02	0.99
DOS	–	Gaze Duration bias UF	− 0.01	0.94

## Discussion

Orthorexia nervosa (ON) is characterised by an excessive preoccupation with healthy eating, manifesting in stringent dietary restrictions and the avoidance of perceived unhealthy foods. Through two distinct studies, we investigated the relative contributions of craving and hedonic motivation for healthy foods versus the preoccupation with foods deemed unhealthy in characterizing ON. Our findings demonstrate that elevated levels of orthorexic features are associated with reduced reward responsiveness and a pattern of attentional avoidance of unhealthy foods. These findings are discussed below.

In Study 1, we measured liking and wanting for healthy and unhealthy foods and orthorexic features. According to Berridge's model [26], two separate but interconnected processes govern motivation: liking, which pertains to hedonic preference or palatability, and wanting, which represents the motivational drive towards a particular food. Liking and wanting can manifest themselves at both explicit conscious and implicit unconscious levels, and interact with homeostatic circuitry to regulate eating behaviour (Berridge et al. 2009). Using explicit ratings, we found that orthorexic features were associated with both lower liking and wanting for unhealthy foods. Additionally, individuals with higher ON scores reported consuming healthy foods more frequently. These findings suggest that the reduced intake of unhealthy foods and the increased consumption of healthy foods typical of ON may be driven by both a diminished motivational drive and a lack of hedonic enjoyment of unhealthy food, which are not perceived as rewarding. This pattern aligns with observations in anorexia nervosa and high-calorie foods (see Lloy and Steinglass 2018, for a review). Notably, anhedonia has been associated with increased food avoidance [39].

In Study 2, we employed an implicit task, i.e. a dot-probe task and recorded eye movements to investigate attentional processes associated with the processing of healthy and unhealthy food in ON. The findings seem to suggest that ON is characterised by a pattern of attentional avoidance of unhealthy foods. First, while individuals with lower ON scores exhibited an attentional bias toward unhealthy foods, consistent with their higher palatability, this pattern was not observed in individuals with higher ON scores. Second, higher ON scores were associated with a greater proportion of initial fixations on unhealthy food items. However, individuals with higher ON scores did not fixate on unhealthy food more than non-food stimuli, unlike those with lower ON scores. These suggest that individuals with higher ON scores show an initial heightened vigilance of unhealthy food cues followed by an attentional shift away from these stimuli. These findings are inconsistent with those of Albery et al. [24], who reported an attentional bias toward healthy food in ON. A potential explanation for this discrepancy could be that, unlike these two studies, we utilised pictorial stimuli. Pictures may indeed be more salient (e.g., [40]), convey more affective information [41], and demonstrate higher ecological validity when compared to words. Importantly, it has been shown that attentional bias outcomes toward food vary depending on whether words or pictures are used, and whether they are high or low-calorie [42]. Interestingly, the attentional pattern that we observed aligns with observations in patients with anorexia nervosa [43–45] and corresponds well with the vigilance-avoidance model, according to which threatening stimuli automatically capture attention at the initial stage but, in later stages, they are avoided as a strategy to reduce arousal and anxiety [46]. Crucially, this pattern may even contribute to reinforcing food restriction in anorexia nervosa [45].

As a matter of fact, the optimal conceptualisation of ON has been debated, with rationales proposed for considering the condition as an eating disorder (e.g. [10]), a form of obsessive disorder (e.g. [11]), or even a form of behavioural addiction (e.g. [13]). Our study suggests that this group exhibits reward system dysregulation specifically toward unhealthy foods, manifested as altered attentional salience. ON is characterised by a pattern of low reward responsiveness to unhealthy foods rather than heightened responsiveness to healthy foods. On a more implicit level, no AB to healthy foods is observed. Instead, the study seems to reveal a pattern of avoidance of unhealthy foods similar to the one observed in anxiety and anorexia [18], which suggests that shared cognitive mechanisms may underlie these disorders. Further studies are required to investigate motivational and attentional alterations in ON. Similarly, more comparisons of the neurocognitive profiles across these pathological conditions would be helpful in further understanding the commonalities between disorders.

Finally, it must be pointed out that the present study has several limitations that warrant consideration. Firstly, reward responsiveness and attentional biases toward food were assessed in two independent samples, with a different proportion of females and males. More studies employing implicit and explicit tasks in the same sample of participants should be performed to clarify whether unhealthy/healthy foods evoke conflicting implicit and explicit evaluations in individuals with ON that differ from those without ON. In addition, our experiments involved individuals with orthorexic features. Although several subjects in the study demonstrated ON on a validated scale, no formal diagnostic criteria for ON currently exist, which limits the generalizability of the findings. Finally, the strong association between liking and wanting suggests that subjective ratings may not sufficiently distinguish between liking and wanting. Future research should therefore employ more objective or complementary methods to dissociate these processes more clearly.

In sum, our observations may suggest the hypothesis that altered motivation and visual orientation may contribute to impaired function in ON. However, future research should investigate whether and how motivational and attentional bias could serve as a potential marker of ON, and how targeting this bias could be applied in treatment interventions.

## Data Availability

The data that support the findings of this study are available from the corresponding author upon reasonable request.
